# Cross-Talk between AURKA and Plk1 in Mitotic Entry and Spindle Assembly

**DOI:** 10.3389/fonc.2015.00283

**Published:** 2015-12-23

**Authors:** Italia Anna Asteriti, Fabiola De Mattia, Giulia Guarguaglini

**Affiliations:** ^1^Institute of Molecular Biology and Pathology, National Research Council (CNR), c/o Department of Biology and Biotechnology, Sapienza University of Rome, Rome, Italy

**Keywords:** kinases, mitosis, G2/M transition, centrosomes, spindle

## Abstract

The Aurora kinase A (AURKA) is involved in different aspects of mitotic control, from mitotic entry to cytokinesis. Consistent with its pleiotropic roles, several AURKA interactors are able to modulate its activity, the best characterized being the microtubule-binding protein TPX2, the centrosomal protein Cep192, and Bora. Bora has been described as an essential cofactor of AURKA for phosphorylation-mediated activation of the mitotic kinase polo-like kinase 1 (Plk1) at the G2/M transition. A complex AURKA/Plk1 signaling axis is emerging, with multiple involved actors; recent data suggest that this control network is not restricted to mitotic entry only, but operates throughout mitosis. Here, we integrate available data from the literature to depict the complex interplay between AURKA and Plk1 in G2 and mitosis and how it contributes to their mitotic functions. We will particularly focus on how the activity of specifically localized AURKA/Plk1 pools is modulated in time and space by their reciprocal regulation to ensure the timely and coordinated unfolding of downstream mitotic events.

## Introduction

About 20 years ago, two loci encoding for serine–threonine kinases required for correct spindle pole assembly were described in *Drosophila* and named “polo” and “aurora” (1–3); these were the forefathers of the corresponding kinase families, now well characterized as key regulators of the cell cycle and mitotic division. Aurora and polo kinases are evolutionary highly conserved, from yeast to mammals (4, 5), and homologs of the originally identified *Drosophila* genes were described in humans as Aurora2 (now AURKA) and polo-like kinase 1 (Plk1), respectively (6–9). Besides the spindle pole phenotypes, several common features led to association of the two kinases, since their discovery. Both display cell cycle-regulated expression (6, 9), with upregulation of mRNAs in the late S and G2 phases ensured by shared transcriptional mechanisms, such as activation by E2F factors (10, 11) and G1-specific repression through CDE/CHR elements (12, 13). Protein levels peak at G2 and mitosis, paralleled by the activation of kinase enzymatic function (9, 14), and drop in a highly coordinated manner at mitotic exit by proteasome-dependent degradation (15). Both kinases localize at centrosomes and spindle poles, although they also display nonoverlapping localization sites, with AURKA associated to spindle pole microtubules, and Plk1 residing at kinetochores; both are also found at the spindle midzone and midbody at ana–telophase (16, 17). Functionally, both AURKA and Plk1 are involved in control of mitotic entry, with an essential role during recovery from DNA damage checkpoint-mediated G2 arrest, and in several aspects of mitotic progression (18–21). Finally, ever since their discovery it has been evident that cancer cells frequently display altered levels of AURKA and Plk1 (7–9, 22) and that downregulating their expression yields antiproliferative effects (23–25); indeed, both kinases are actively studied as potential anticancer targets (26, 27). All these similarities suggested direct links between AURKA and Plk1, which started to come out only in the last 10 years. Here, we review data about the interplay of AURKA and Plk1, focusing on the emerging view of how this can contribute to AURKA activation at distinct subcellular sites and in different cell cycle windows, thus finely coordinating downstream mitotic events.

## Activation Mechanisms for AURKA and Plk1

Phosphorylation of a threonine residue within the activation loop of AURKA and Plk1 kinases, Thr-288 and Thr-210, respectively, is crucial for their enzymatic activity (28, 29). Phosphorylation of Plk1^Thr-210^ occurs upon release of an inhibitory intramolecular interaction between the N-terminal catalytic domain and the C-terminal “polo-box” domain (PBD). The latter is a phosphoserine/threonine recognition domain; its binding to target phosphopeptides, mainly generated by the cdk1 kinase, impairs the interaction with the catalytic domain, thus triggering Plk1 activation (30, 31). Plk1 activation mechanism, thus, relies on making the region where Thr-210 lies accessible; Thr-210 can then be phosphorylated by an upstream kinase (see the following sections).

Data collected so far indicate a more complex mechanism for AURKA activation. AURKA^Thr-288^ lies within an AURKA consensus motif and is therefore regarded as an autophosphorylation site. It is still debated whether autophosphorylation is achieved by an intra- or intermolecular reaction, and conformational shifts as well as dimerization appear to underlie different activation states (32–34). Indeed, data in the literature indicate multiple binding partners (see the following sections) that are able to stimulate AURKA activity without a direct enzymatic action but rather by inducing specific conformational transitions. These observations suggest that cells need to manage distinct pools of AURKA, acting at distinct subcellular sites and displaying different extents of activity.

Interestingly, although activation mechanisms for AURKA and Plk1 are distinct, coupling intracellular localization with function appears to be a conserved feature: for Plk1, the PBD is also required for correct targeting of the kinase to centrosomes, kinetochores, and spindle midzone (35, 36), and the major AURKA activators, namely Cep192 and TPX2, mediate AURKA binding to centrosomes and microtubules, respectively (37–39).

## The AURKA/Plk1/Bora Axis and Mitotic Entry

**Figure 1:**
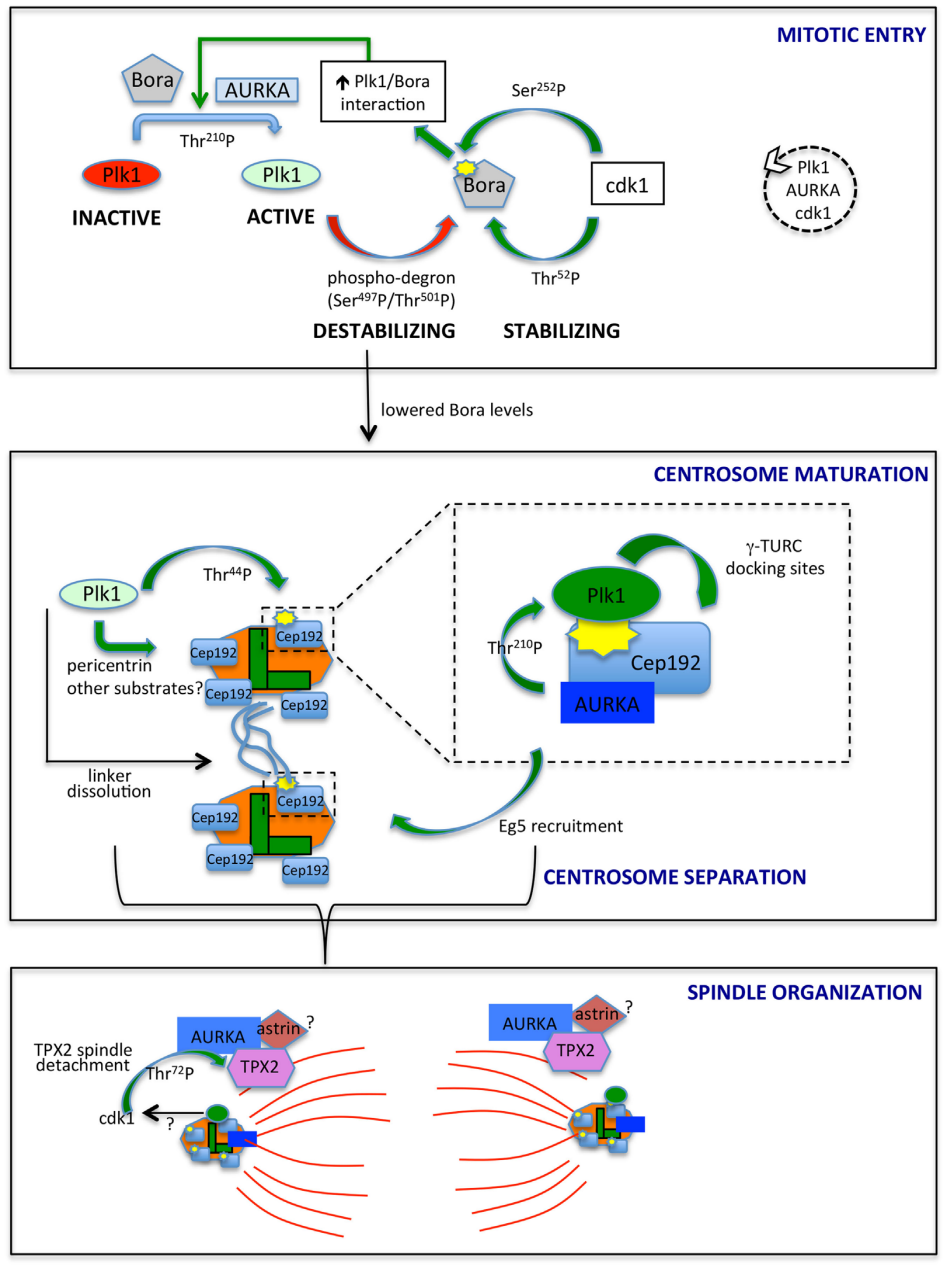
**AURKA and Plk1 in mitotic entry and spindle formation**. The best characterized links between AURKA and Plk1 are schematized. In mitotic entry (upper box), the combined action of AURKA and Bora activates Plk1, while antagonistic phosphorylation events by Plk1 and cdk1 control Bora stability. The dashed circle on the right indicates the ongoing feedback loop leading to the activation of Plk1, AURKA, and cdk1. Lowered Bora levels enable the interaction of AURKA with Cep192 (central box) and TPX2 (lower box), at centrosomes (centrioles, green; PCM, orange) and microtubules (red), respectively. The enlargement in the central box depicts the scaffolding function of Cep192, leading to recruitment of AURKA and Plk1, activation of the latter and generation by activated Plk1 of γ-TURC-docking sites, with consequent centrosome maturation. Note that Cep192-bound AURKA is activated in a dimeric form, although not represented here to simplify the scheme. Cep192/Plk1/AURKA also contributes to centrosome separation via Eg5 recruitment, and Plk1 independently participates to this process by triggering centrosome linker (light blue lines) dissolution. Separated centrosomes nucleate spindle microtubules that are organized, among others, by AURKA/TPX2 complexes, possibly bound to astrin (lower box). cdk1 phosphorylation of TPX2, possibly influenced by Plk1 activity, yields decreased binding to microtubules. Centrosomal proteins in the lower panel are schematized as in the upper ones, although for space reasons their names are not indicated. The yellow symbols identify PBD-docking sites. Green arrows indicate positive regulatory events, while red arrows represent negative ones. Phosphorylated residues or domains are indicated on the arrows. The different intensities of colors for Plk1 and AURKA denote a different extent of activity.

The direct link between AURKA and Plk1 came with the identification of AURKA as the upstream kinase responsible of phosphorylation of Thr-210 in the Plk1 activation loop, an event requiring the presence of the coactivating protein Bora (19, 40) (Figure 1, upper box). Distinctly from other AURKA activators, Bora does not modify AURKA activity *per se* but rather interferes with the intramolecular interaction between the catalytic domain of Plk1 and the PBD, so to render Thr-210 accessible (40). Consistently, Bora does not significantly increase AURKA activity toward substrates other than Plk1 (19, 40), and the extent of activation of AURKA coimmunoprecipitated with Bora, as assessed by p-Thr-288, is by far lower than that associated with the fractions immunoprecipitated with TPX2 or Cep192 (41, 42). Although low, this activity may suffice to trigger what was defined as the “outer feedback loop” through which AURKA, Plk1, and cdk1 activate each other (43). Phosphorylation of Bora at Ser-252 (human) by cdk1 creates a PBD-docking site and promotes Bora/Plk1 interaction (Figure 1); consistently, phosphorylation of Bora by cdk1 enhances its ability to stimulate AURKA-mediated Plk1 activation (41, 44). A second residue on human Bora, i.e., Thr-52, is responsive to cdk1: GST-tagged human Bora carrying Thr-52 substitution to alanine is destabilized in CSF-arrested *Xenopus* oocytes extracts (45), thus suggesting that cdk1 phosphorylation plays also a role in protecting Bora from degradation. An opposite effect is mediated by Plk1 in that Plk1 phosphorylation of Bora in the 496-DSGYNT-501 degron triggers Bora degradation through the SCF-β-TrCP pathway (41, 46) about 2 h before mitotic entry (45, 47). Consistently, a decreased interaction between Plk1 and Bora, by mutating the previously mentioned Bora^Ser-252^ to Alanine, influences Bora stability: (i) it prevents GST-Bora degradation in CSF extracts (45) and (ii) in human cells, it impairs the interaction between Bora and β-TrCP (41). In addition, it prevents Bora accumulation induced – as a result of a dominant-negative effect – by kinase-dead Plk1 (41). The opposite effects of cdk1-mediated phosphorylation of Bora on Thr-52 and Ser-252 suggest that timely degradation of Bora constitutes a strictly controlled event; the balance between phosphorylation of Thr-52 by cdk1 and on the degron sequence by Plk1 may determine when the switch toward SCF-β-TrCP-mediated degradation of Bora occurs (Figure 1).

### The Spatiotemporal Level of Bora/AURKA/Plk1 Regulation

**Figure 2:**
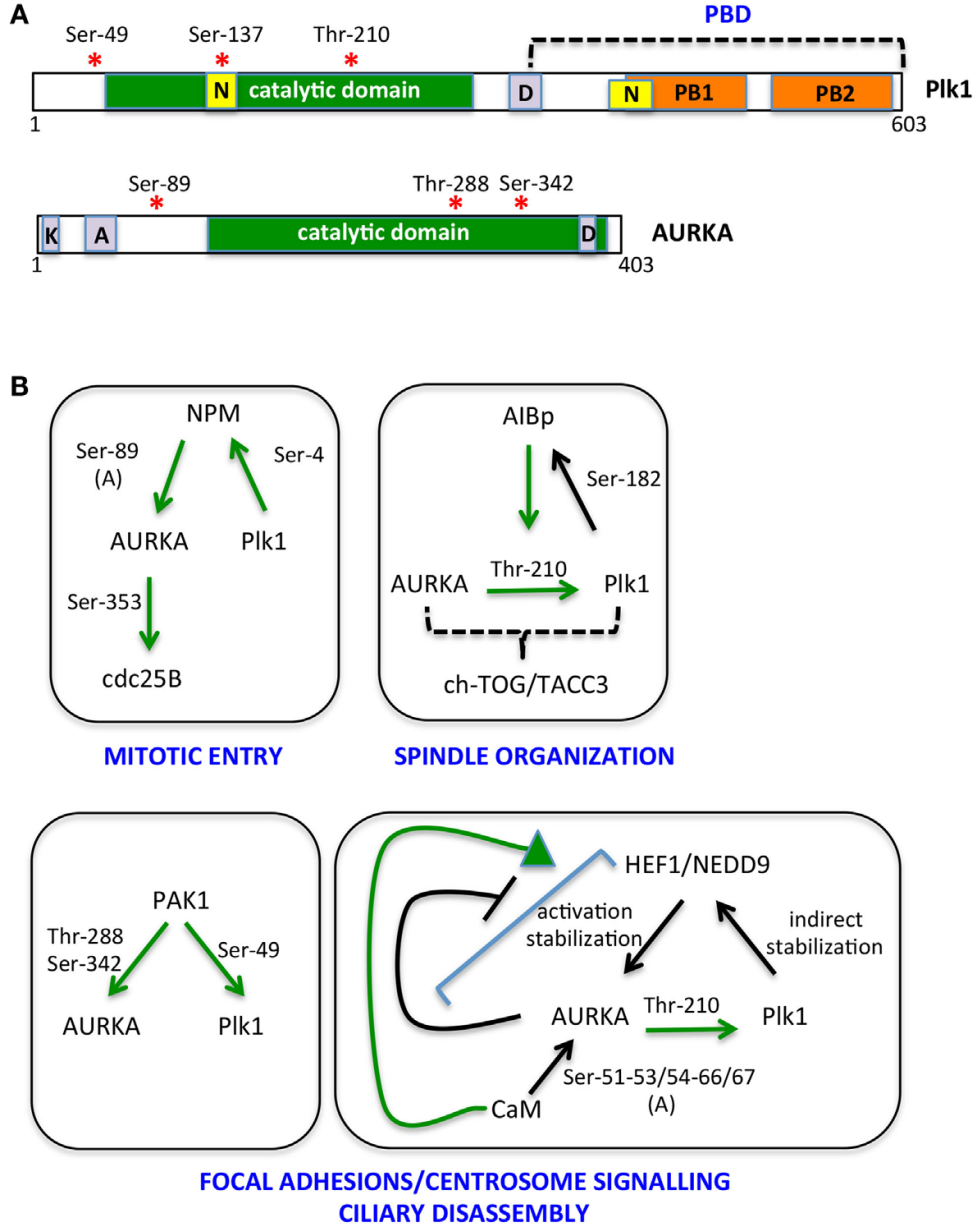
**The multiple AURKA and Plk1 activating networks**. **(A)** A schematic representation of Plk1 and AURKA kinases is shown with activating phosphorylation sites indicated by red asterisks. Yellow boxes represent nuclear localization sequences (N); orange ones are polo boxes (PB1 and 2); and violet ones are degradation motifs (D, destruction box; K, KEN box; A, A box). Catalytic domains are in green. **(B)** Networks involving NPM, AIBp, PAK1, HEF1/NEDD9, and calmodulin (CaM) as activators and/or substrates of AURKA and Plk1 are represented. Green arrows indicate direct and clarified activating events; phosphorylated residues are indicated; and **(A)** denotes induction of an autophosphorylation event. Downstream substrates and proposed regulated processes are included.

The bulk of cycB1/cdk1 complexes is cytoplasmic until prophase, when it promotes its own translocation to the nucleus (48, 49). On the other hand, although Thr-210-phosphorylated Plk1 is first detected at centrosomes, results obtained using a FRET biosensor suggest that Plk1 kinase activity first increases in the nucleus and raises in the cytoplasm only 2 h before mitotic entry (19, 45, 47), at a time that coincides with the onset of Bora degradation (45, 47). Together with the recent observation that Bora is prevalently cytoplasmic in mammalian cells (47), these data suggest that cdk1 and Plk1 activities antagonistically modulate Bora levels, with cdk1-mediated Thr-52 phosphorylation protecting Bora from degradation until cytoplasmic Plk1 activity raises. A potential player in this regulatory mechanism is the peptydylprolyl isomerase Pin1, a modulator of the G2/M transition, which promotes Bora degradation (50) and whose activity and stability are controlled by AURKA and Plk1, respectively (50, 51); further studies are needed to understand how these molecular events interplay in regulating mitotic entry. Phosphatases acting both on kinases themselves and on their substrates, with time- and space-dependent selectivities (52), are also expected to play a role in this fine-tuned regulation. The key serine–threonine phosphatases that counteract mitotic kinase activity are PP1 subunits and PP2A complexes (53, 54). Potentially relevant to Bora degradation, PP2A activity, which is able to counteract Plk1 and cdk1 substrate phosphorylation, is inhibited in the cytoplasm by the Mastl/Greatwall kinase before mitotic entry (52). Translocation of nuclear Greatwall to the cytoplasm is promoted by both cdk1 and Plk1 (55, 56): this mechanism may ensure that phosphorylation of Plk1 cytoplasmic substrates, such as Bora, only accumulates subsequent to Plk1 activation in the nucleus and to cdk1 nuclear import. Whether a differential specificity of action of phosphatases on the different Bora residues phosphorylated by cdk1 and Plk1 exists is an open question that may provide further hints on the time-dependent regulation of Bora stability. As also recently proposed by Bruinsma and colleagues (47), differentially localized phosphatase activity may generally contribute to time-dependent compartmentalization of Plk1 activity, thus explaining why the latter is first observed in the nucleus, although Bora is reported to be strictly cytoplasmic and the extent and timing of AURKA nuclear entry is poorly characterized. We also noticed that the NLS sequences described for Plk1 fall within the catalytic and polo-box domains (57, 58) (Figure 2), raising the possibility that formation of import complexes in the cytoplasm impairs Plk1 kinase function, which would be only released in the nucleus. Modulated interaction between Plk1 and importins may therefore contribute to the switch to cytoplasmic Plk1 activity 2 h before mitotic entry: indeed Ser-137 within one of the NLS sequences (Figure 2) is phosphorylated *in vivo* and this is described as an activating event for Plk1, although so far described only in late mitosis (29, 59). Alternatively, over time, increased cdk1-generated PBD-docking sites on Plk1 cytoplasmic substrates could retain Plk1 in the cytoplasm by competing with importins for Plk1 binding.

### Changing Interactors for Progressing Through Mitosis

What is the functional significance of Bora degradation before mitotic entry by the same protein (Plk1) that it activates? A possible explanation is that the cdk1/AURKA/Plk1 signaling cascade generating the mitotic entry signal (43) must timely switch toward other pathways to sustain spindle assembly and mitotic progression. Evidence summarized below supports the notion that lowering Bora levels is necessary to make AURKA available to other partners. Immunoprecipitation experiments indicate that AURKA complexes containing Bora or TPX2 are distinct and that artificially increasing Bora levels – through Plk1 inactivation – changes the stoichiometry and decreases the amount of TPX2 bound to AURKA (41). In addition, AURKA localization to spindle poles, mediated by Cep192 and TPX2 (see below), is altered when Bora levels are increased by overexpression or by Plk1 inactivation (41). This is likely accounting for the proposed role of Plk1 in AURKA centrosomal localization (38, 60) and further indicates that the Bora/Plk1 complex is able to compete with other AURKA activating/localizing partners.

Together, these observations suggest that AURKA activity initially needs to be focused toward the Plk1 kinase; this activates the AURKA–Plk1–cdk1 loop, until a threshold is reached and the cell is committed toward mitosis (43, 61). Now AURKA and Plk1 kinases must be properly redirected toward their mitotic activators and substrates to coordinate mitotic entry with centrosomal and spindle processes (Figure 1). How does Plk1 remain active in mitosis when Bora is degraded? On the one hand, the accessibility of Thr-210 may not represent a limiting factor in mitosis, when high cdk1 activity creates abundant PBD-docking sites. On the other hand, recent data indicate that although Bora levels are strongly reduced in mitosis, a residual fraction exists (45), and it is responsible of Plk1 activation throughout the division process (62). An independent protein, Furry, has been described to activate Plk1 through AURKA, with a mechanism comparable to Bora (63). It will be interesting to investigate whether this redundancy underlies subcellular, temporal, or cell-type specificity. Most importantly, Cep192 emerging scaffolding functions may bypass the requirement for Bora in the AURKA/Plk1 axis at centrosomes.

## The AURKA/Plk1/Cep192 Axis Controls Centrosome Maturation and Separation

The drop in Bora levels following Plk1 activation may ensure that centrosomal processes leading to spindle assembly, depending on other AURKA containing complexes, start only when the mitotic entry signaling cascade is fully active. The centrosomal protein Cep192, involved in both centrosome maturation and separation (39, 64), appears as a key coordinator of AURKA and Plk1 activity at this stage. Cep192 was first shown to trigger dimerization-driven AURKA activation at centrosomes in *Xenopus* egg extracts (65) and was later confirmed as a key AURKA centrosomal activator in mammalian cells (42, 66). Cep192-bound AURKA is highly active compared to the Bora- or TPX2-bound pools (42, 65). In human cells, the interaction between AURKA and Cep192 is reported from S phase (42); the strong increase in centrosomal Cep192 at mitotic entry, just before centrosome separation (39, 64), suggests that more Cep192–AURKA centrosomal complexes exist at this stage, in agreement with the proposed requirement of freeing AURKA from Bora-containing complexes. Importantly, Plk1 has recently been shown to be a part of the AURKA/Cep192 axis driving centrosome maturation (Figure 1, central box): Cep192 acts as a scaffold for both Plk1 and AURKA and is the key recruiting factor for the kinases at centrosomes, with Plk1 binding following that of AURKA (39, 42, 66). Cep192 brings AURKA and Plk1 in close proximity thereby enabling Plk1^Thr-210^ phosphorylation (42, 66). AURKA-activated Plk1 creates its own PBD-docking site on Cep192 by phosphorylating Cep192^Thr-44^ (42, 66); a subsequent AURKA-independent PBD-docking site centered on Cep192^Ser-995^ has been reported (42), although the separation of the functional roles of Thr-44 and Ser-995 needs further investigation.

It could be speculated that preceding activation by Bora/AURKA generates the low Plk1 activity required for initial phosphorylation of Thr-44, while ensuing stabilization of Cep192/Plk1/AURKA complexes (42), where AURKA activity is higher, boosts the signaling cascade leading to centrosome maturation. Plk1 is required for Cep192 centrosomal localization, partly through phosphorylation of pericentrin (67, 68), supporting the hypothesis that an initial Cep192-independent Plk1 activation triggers a subsequent and more sustained Cep192-mediated one. Cep192/AURKA-activated Plk1 in turn phosphorylates Cep192 to generate γ-TURC-docking sites and induce the sudden increase in pericentriolar material (PCM) characterizing centrosome maturation (42, 66) (Figure 1, central box).

Centrosome separation requires linker dissolution and Eg5-mediated centrosome movement, both involving Plk1 (69–71). While linker dissolution does not require Cep192, the observation that loss of Cep192 impairs Eg5 centrosomal localization and centrosome separation (39, 66) suggests that the role of Plk1 in centrosome movement passes through the Cep192/AURKA axis, with a key upstream involvement of centrosomal cyclin B2/cdk1 (72) (Figure 1, central box).

Cep192 complexes identify an AURKA pool clearly distinct from the microtubule- and TPX2-bound one: (i) the AURKA/Cep192 interaction occurs also in the absence of microtubules (42); (ii) Cep192 and TPX2 bind to the same region of AURKA and are detected in independent AURKA complexes (65); (iii) Cep192-loaded beads recapitulate in CSF-arrested *Xenopus* oocytes extracts the functions as microtubule-organizing center (MTOC) of AURKA-loaded beads but not their ability of RanGTP-induced spindle organization (66, 73). TPX2 is a RanGTP-regulated factor (74); these observations together suggest that the pools of AURKA bound to Cep192 and TPX2 are functionally separated and involved in centrosome maturation and spindle assembly, respectively. The observation that both Cep192/AURKA and TPX2 regulate Eg5 activity (66, 75) may reflect independent functions in centrosome separation or an interplay of the two pools of AURKA in this process yet to be unveiled.

## Microtubule-Associated AURKA Pools and Spindle Organization

Microtubule-organizing functions of AURKA are less obviously linked to Plk1 activity. AURKA localization to microtubules is mediated by the microtubule-binding protein TPX2 (37, 38), which also activates AURKA by stabilizing the active conformation and making AURKA^Thr-288^ inaccessible to the PP1 phosphatase (76). In addition, TPX2 protects AURKA from APC/C^Cdh1^ proteasome-dependent degradation in G2 and early mitosis, with TPX2 depletion impairing accumulation of high levels of AURKA in prometaphase (77). *Xenopus* Plx1 has been shown to phosphorylate TPX2 on Ser-204, with a positive effect on TPX2-mediated AURKA activation (78). A corresponding mechanism has not been explored in mammalian cells given the poor conservation of the phosphorylated site. Yet, phosphoproteomic screenings identified TPX2 *in vivo* phosphosites that are likely to be phosphorylated by Plk1 (79, 80). Furthermore, TPX2 abnormally accumulates at spindle poles in Plk1-interfered mitoses (38), and recent data show that cdk1-mediated phosphorylation of TPX2^Thr-72^ negatively modulates TPX2 association to the mitotic spindle (81). It is, therefore, conceivable that Plk1 activity at mitotic centrosomes, through its effects on cdk1, influences TPX2 mobility at spindle poles (Figure 1, lower box).

Astrin is an independent regulator of AURKA localization at microtubules, with no effect on the kinase activity (82); RNA interference-mediated depletion of astrin induces spindle defects reminiscent of those observed following AURKA inactivation (82, 83). Astrin localization to the spindle is in turn mediated by TPX2 (82). Interestingly, Plk1 has also been detected in astrin–kinastrin complexes in mitotic cell extracts (84).

Together these observations suggest that exploring the interplay between AURKA, TPX2, Plk1, and astrin deserves further investigation and may improve our understanding of AURKA spindle-organizing functions.

## The Growing Network of AURKA Activators

Additional activators of AURKA at centrosomes and spindle poles have been described, many being also functionally linked to Plk1 (Figure 2).

Nucleophosmin (NPM) activates AURKA by stimulating a newly identified autophosphorylation event, on Ser-89 (85). Phosphorylation of NPM by Plk1 is required for its mitotic functions (86), while NPM depletion does not affect Plk1^Thr-210^ phosphorylation (85). These observations suggest that NPM-activated AURKA is generated when Plk1 activation has become prevalently AURKA independent; alternatively, since the only AURKA substrate affected by NPM depletion is so far CDC25B, NPM may provide AURKA specificity of action toward a limited set of substrates.

The AIBp protein, colocalizing with AURKA at centrosomes and spindle poles, has recently been reported as an AURKA regulator, relevant for Plk1 activation and in turn a substrate of it; the observation that localization of the downstream AURKA targets TACC3 and ch-TOG is affected by AIBp depletion, while PCM recruitment is not, together with the associated spindle pole phenotypes, suggest an involvement of AIBp in the spindle-organizing functions of AURKA (87).

AURKA activators also include proteins that localize both at focal adhesions and centrosomes, in particular the PAK1 kinase and the HEF1/NEDD9 scaffolding protein (88, 89). PAK1 promotes AURKA activation by directly phosphorylating Thr-288 and Ser-342 (89) and also phosphorylates Plk1^Ser-49^, an event that contributes to its activation (90). HEF1/NEDD9 promotes the catalytic activity of AURKA (88) and also stabilizes it (91); the interaction between AURKA and HEF1/NEDD9 is favored by CaM (92), while it is inhibited by AURKA phosphorylation of HEF1/NEDD9 (88), indicating the presence of a negative feedback loop. Plk1 in turn indirectly regulates HEF1/NEDD9 stability, with deriving increased AURKA activity signaling back on Plk1 activation (93). The focal adhesion localization of PAK1 and HEF1/NEDD9 suggests that they define a pool of AURKA responsible of a signaling path that links loss of cell adhesion – typical of the cell division process – with mitotic centrosomal events and mitotic entry (94, 95). This pool appears also to be involved in the non-mitotic role of AURKA in cilia disassembly at cell cycle reentry from G0 (92, 96), a process that also requires Plk1 activity (93). An additional interactor of AURKA involved in both cell–cell adhesion and cell proliferation and survival (97) is Ajuba. The interaction between AURKA and Ajuba was first described in human cells (98), where it was shown as a key AURKA-activating step at G2 centrosomes (98). Recent data suggest that the activation mechanism relies on the ability of Ajuba, upon binding to AURKA N-terminus, to prevent an inhibitory intramolecular interaction between the N- and C-termini of the kinase (99); in addition, the subsequent binding of a distinct Ajuba domain to the C-terminus of AURKA directly stimulates kinase activity (99). A role of Ajuba in AURKA regulation has been confirmed in *Drosophila* neuroblasts, although data indicate an effect on localization, rather than activation, of the kinase (100). Organism and/or cell-type specificity may account for the observed differences, although cell cycle- (G2 vs. mitosis) or reporter- (phospho-AURKA/phospho-H3 vs. phospho-TACC3) dependent effects may also be envisaged.

## Conclusion

Several AURKA activators have been described at centrosomes and microtubules, and evidence exist that they create independent complexes with the kinase. The scaffolding functions of some of them and the finding of specific phospho-AURKA fractions depending on the bound activator suggest that distinct interactors define specific AURKA pools with differential kinase activity and/or substrate specificity. More interconnected analyses of the different AURKA pools and a better spatiotemporal resolution of their formation during the cell cycle are expected to uncover in the next years how they ensure tight coordination of downstream events. Plk1 is a key substrate of AURKA and at the same time a major regulator of the multiple AURKA activators: besides contributing to generate an activation feedback loop that reinforces AURKA and Plk1 activities at mitotic entry, this is also emerging as a mechanism to impart time-dependent regulation to the unfolding of AURKA-regulated events. Exploring the contribution of the AURKA/Plk1 axis in mitotic control, including in newly identified mitotic functions of AURKA (101–104), is a promising field of investigation for the future.

## Author Contributions

IAA critically analyzed the literature, contributed to article writing, and prepared figures. FDM critically analyzed the literature and discussed extensively review structure, contents, and models. GG conceived the structure of the review, critically analyzed the literature, and wrote the article.

## Conflict of Interest Statement

The authors declare that the research was conducted in the absence of any commercial or financial relationships that could be construed as a potential conflict of interest.
